# Multi-Feature Automatic Extraction for Detecting Obstructive Sleep Apnea Based on Single-Lead Electrocardiography Signals

**DOI:** 10.3390/s24041159

**Published:** 2024-02-09

**Authors:** Yu Zhou, Kyungtae Kang

**Affiliations:** 1 Department of Computer Science and Engineering, Major in Bio Artificial Intelligence, Hanyang University, Ansan 15588, Republic of Korea; zhouyu@hanyang.ac.kr; 2 Department of Artificial Intelligence, Hanyang University, Ansan 15588, Republic of Korea

**Keywords:** obstructive sleep apnea, diagnosis, continuous wavelet transform, Gramian angular field, hybrid dataset, convolutional neural network, automatic feature extraction

## Abstract

Obstructive sleep apnea (OSA), a prevalent sleep disorder, is intimately associated with various other diseases, particularly cardiovascular conditions. The conventional diagnostic method, nocturnal polysomnography (PSG), despite its widespread use, faces challenges due to its high cost and prolonged duration. Recent developments in electrocardiogram-based diagnostic techniques have opened new avenues for addressing these challenges, although they often require a deep understanding of feature engineering. In this study, we introduce an innovative method for OSA classification that combines a composite deep convolutional neural network model with a multimodal strategy for automatic feature extraction. This approach involves transforming the original dataset into scalogram images that reflect heart rate variability attributes and Gramian angular field matrix images that reveal temporal characteristics, aiming to enhance the diversity and richness of data features. The model comprises automatic feature extraction and feature enhancement components and has been trained and validated on the PhysioNet Apnea-ECG database. The experimental results demonstrate the model’s exceptional performance in diagnosing OSA, achieving an accuracy of 96.37%, a sensitivity of 94.67%, a specificity of 97.44%, and an AUC of 0.96. These outcomes underscore the potential of our proposed model as an efficient, accurate, and convenient tool for OSA diagnosis.

## 1. Introduction

Obstructive sleep apnea (OSA) is a widely recognized respiratory disorder marked by recurrent obstruction and collapse of the upper airway during sleep, resulting in periodic hypoxia. In its severe manifestation, OSA is implicated in the etiology of several comorbidities, including, but not limited to, systemic hypertension, coronary artery disease, cardiac arrhythmias, and cerebrovascular pathologies [1]. Characteristically, subjects suffering from OSA exhibit more than 30 episodes of apnea per seven-hour sleep cycle. Each episode is defined by a cessation of nasal and oral airflow for a duration exceeding ten seconds, constituting a clinical manifestation recognized as a complete apnea event.

Sleep apnea (SA) manifests in three distinct forms: obstructive sleep apnea (OSA), central sleep apnea (CSA), and mixed sleep apnea (MSA), each distinguished by characteristic respiratory patterns [2]. OSA results from physical obstruction of the upper airway, whereas CSA originates from the brain’s inability to transmit the requisite signals to the respiratory muscles. Globally, OSA affects approximately one billion individuals [3]. The dataset utilized in this study predominantly focuses on OSA, thereby directing the primary emphasis of this research towards the classification of OSA.

If not addressed, OSA can precipitate a range of health complications, encompassing disturbances like snoring-induced awakenings, migraines, afternoon lethargy, cognitive concentration challenges, memory deficits, and psychological issues, including anxiety and depression [4]. Polysomnography (PSG) stands as the gold standard for OSA diagnosis, encompassing the monitoring of various physiological parameters, such as airflow, respiratory effort, electroencephalography (EEG), electrocardiography (ECG), and oxygen saturation (SaO_2_) during sleep [5]. However, the precision of PSG is counterbalanced by its demands for extensive time and specialized expertise, presenting hurdles in terms of financial cost, time consumption, and potential patient discomfort. Creating accurate, affordable, and easily accessible technology for identifying and tracking sleep events is essential in lessening cardiovascular, psychiatric, and various other health risks associated with sleep apnea. Recent advancements in single-channel signal analysis, including the use of ECG, SaO_2_, and respiratory signals, are being pursued to reduce diagnostic expenses and improve user-friendliness [6,7,8,9,10]. The utilization of ECG is particularly noteworthy due to its pivotal role in OSA pathology and its compatibility with wearable technology applications. While multi-lead ECGs offer a comprehensive view of cardiac activity, capturing heart functions and potential anomalies more thoroughly, single-lead ECGs hold advantages for long-term continuous monitoring essential for the early detection and management of OSA. Particularly in the realm of home medical monitoring, single-lead ECGs stand out for their portability and lower cost. By employing refined algorithms and feature extraction methods, it is possible to somewhat offset the informational limitations inherent in single-lead signals.

Initially, traditional machine learning algorithms were employed for OSA detection. Nevertheless, given the intricacy of physiological signals and the constrained extraction capabilities inherent in these algorithms, recent scholarly attention has pivoted towards sophisticated deep learning models. Leveraging the advancements in computational prowess, deep learning algorithms have outperformed their traditional machine learning counterparts in autonomously extracting significant features. Several automated OSA detection methodologies have been introduced to alleviate the technical and economic constraints associated with conventional PSG. Research indicates a close correlation between OSA events and signal fluctuations, involving variations in heart rate variability (HRV), alterations in the morphology of ECG signals, and changes in the duration of the QRS complex [11]. Experiments have shown that algorithms integrating morphological variability features tend to yield superior results [12]. Furthermore, in the process of OSA detection, the focus is typically placed on the characteristics of HRV.

Drawing on a multimodal strategy, data from multiple sources can sometimes complement each other, revealing patterns not visible when using single data types in isolation. This approach assists in generating more reliable predictions. In the deep learning field, multimodality refers to the use of diverse forms of information as inputs for deep learning models [13]. This study proposes the multi-feature automatic extraction for detecting OSA (MFAE-OSA) method. Addressing the issue of insufficient generalization capabilities of a single model for multiple features [14], this study proposes the utilization of a hybrid model for the extraction of multiple features, achieving high-precision detection when multiple features are inputted. The contributions of this research are as follows:

(1) Utilization of a range of input data and application of continuous wavelet transform (CWT) to produce scalogram images from ECG signals that highlight HRV features. In addition to HRV, the research also uses the Gramian angular field (GAF) technique to convert ECG signals into images that reflect temporal features. This methodology serves to expand the feature set and enrich the input data. Such integration of various techniques offers new avenues for feature selection in the detection of OSA, enhancing the multidimensional analysis capabilities of the study;
(2) A hybrid ensemble CNN model is designed for OSA detection. The model integrates both residual and inception architectural frameworks and employs a soft voting mechanism with gentle weighting for evaluation. By utilizing diverse model architectures for automatic feature extraction and enhancement, the system is capable of concurrently processing multi-feature datasets. This approach eliminates the need for manual feature extraction, thereby reducing the potential for human error;
(3) The proposed method has the potential to be accurate and efficient for diagnosing OSA. The findings indicate that the suggested classifier can attain an accuracy of 96.37%, a sensitivity of 94.67%, a specificity of 97.44%, and an AUC of 0.96 on the hybrid image datasets.

The rest of this article is organized as follows: Section 2 presents the related work. Section 3 describes the dataset and preprocessing. Section 4 presents the materials and methods. Section 5 describes the experiment and result. Finally, we discuss and conclude the paper in Section 6.

## 2. Related Work

Over the past few decades, numerous techniques for identifying OSA through ECG signals have been developed. According to Ucak et al. [15], apneic episodes are related to changes in the RR interval in the ECG signal. Valavan et al. [16] presented a detection method that utilizes support vector machines (SVMs) employing a grid search algorithm. This technique is trained using features derived from ECG data, specifically focusing on heart rate variability (HRV). Viswabhargav et al. [17] proposed that employing a sparse residual entropy feature within a radial basis function (RBF) kernel-based support vector machine (SVM) classifier enhances performance, as evidenced by the recorded accuracy, sensitivity, and specificity values of 78.07%, 78.01%, and 78.13%, respectively. Sharma et al. [12] used Hermite expansion coefficients for the analysis of one-minute ECG signals and applied a least-squares support vector machine (LS-SVM) classifier equipped with a Gaussian radial basis function (RBF) kernel. This approach resulted in an achieved accuracy of 83.4%. Kunyang et al. [18] introduced a model for sleep apnea (SA) classification based on neural networks (NNs) integrated with hidden Markov models (HMMs). Their framework employed a hybrid approach combining sparse autoencoders, neural networks, and HMMs. The classification accuracy of this model for detected apnea events reached 84.7%. However, it is noted that stacked sparse autoencoders primarily serve as an unsupervised feature transformation mechanism and may not effectively extract features.

Tripathy et al. [19] proposed an innovative method for the analysis of cardiopulmonary (CP) signals, integrating fast adaptive bivariate empirical mode decomposition (EMD) with crossing time–frequency analysis. This approach constructs the CP signal by amalgamating the heart rate (HR) and respiratory rate (RR) components derived from the ECG signal. By employing a combination of the SVM and the random forest classifiers within a 10-fold cross-validation setup, their approach resulted in average sensitivity and specificity rates of 82.27% and 78.67%, respectively.

A multitude of ECG signal attributes utilized for classification in these methodologies can be ascertained through manual determination. The extraction of the QRS complex waves is predominantly conducted using manually selected features. Predominantly, contemporary approaches depend on nonlinear attributes sourced from physiological data, alongside frequency and time domain representations, necessitating considerable expertise and interpretative knowledge. This often necessitates extensive manual preprocessing. Addressing this, Wang et al. [20] proposed an approach employing an augmented LeNet-5 convolutional neural network (CNN) for OSA classification, automating the feature extraction and reaching 87.6% accuracy.

Advancements in image-centric research have markedly amplified the application of deep learning in the realms of medical imaging and signal processing. Deep neural networks (DNNs) have demonstrated efficacy in the field of ECG analysis. In [21], the study utilized spectrogram signatures obtained from ECGs through the fast Fourier transform (FFT) for classifying OSA, achieving a detection accuracy of 92.6%. In [22], the authors presented a method for detecting OSA using a DNN that processes ECG scalograms created through wavelet transform, attaining a per-minute class accuracy of 86.22%. Using fused scalogram and spectrogram images, Niroshana et al. [23] proposed that 2D-CNNs can perform OSA detection on fused spectral images with, on average, 92.4% accuracy, 92.3% recall, and 92.6% specificity.

Although fusion image methods have been proposed, these predominantly focus on the heart rate variability (HRV) characteristics post-image transformation. In contrast, our method extends beyond this focus by incorporating Gramian angular field (GAF) matrix images, which encapsulate temporal characteristics, thereby enriching the feature set. This approach offers a multifaceted perspective for OSA detection by providing a diverse set of features. Moreover, while previous studies often rely on single-model approaches, our proposed hybrid ensemble model is capable of processing different features simultaneously. Furthermore, the accuracy of this integrated learning approach is expected to surpass that of individual classifiers due to the collective performance enhancement.

## 3. Dataset and Preprocessing

### 3.1. Dataset

For the purpose of obtaining dependable outcomes, our study utilized the PhysioNet Apnea-ECG database (available at: https://physionet.org/content/apnea-ecg/1.0.0/ accessed on 1 January 2024), publicly made accessible by Philipps University [24]. Table 1 delineates the characteristics of single-lead ECG signals as found in the database:

The dataset includes specialist annotations for every one-minute interval of the ECG signal [24,25]. Each segment of signals was labeled and classified as either “normal breathing” (*N*) or “impaired breathing” (*A*), without differentiating hypopnea.

### 3.2. Signal Preprocessing

ECG signals gathered from monitoring frequently encounter different types of noise interference. To enhance analytical quality, these signals necessitate denoising. Standard practice involves filtering the signal to remove unwanted frequency components, maintaining its intrinsic structure. The amplitude range of unprocessed, noisy ECG signals spans from −2 V to +2 V. In signal processing, if the filter order is insufficient, there is a risk of losing parts of the output signal. In scenarios where the cutoff frequency is set too low, some ECG signals may become irretrievable. For mitigating high-frequency disruptions, ECG signals were filtered using a fourth-order Butterworth low-pass filter, set at a cutoff frequency of 40 Hz [26]. Baseline drift, characterized by the deviation of the signal’s base axis, alters its normal baseline trajectory. Muscle noise, often generated during physical exertion, can similarly impact signal integrity. In medical settings, the use of a series of leads and adhesive electrode patches is essential, generally affixed to predetermined sites on the patient’s torso, arms, and legs. These electrodes are adept at detecting the subtle electrical currents emanating with each cardiac cycle, thus facilitating the acquisition of the patient’s ECG data. The interaction between the electrodes and the patient’s movements can introduce muscle noise into these data due to the physical connectivity and motion. To combat this challenge, contemporary methodologies have been developed to diminish muscle noise interference through specific advancements in material science. An example is the development by J. Cao et al. [27] of a nano-LM-based, highly durable, and stretchable electrodes, designed for the prolonged dynamic monitoring of human health. Signal preprocessing techniques are also applied as a strategy to reduce noise impact. Owing to the availability of public data, this study employs filtering techniques to reduce the impact of noise. To address these issues, a bandpass filter, specifically a fourth-order filter with a passband of 0.5 Hz to 15 Hz, was employed [28]. Considering the presence of biologically implausible data points, a median filter was utilized to eliminate isolated noise peaks and align adjacent values more closely with the true signal [29]. Additionally, due to individual variations in ECG signals, a *z*-score function was applied to standardize, facilitating more consistent analysis. This function is defined as Equation (1):

(1) $$z=\frac{x-\mu }{\sigma },$$

where μ represents the mean and σ denotes its variance.

**Figure 1:**
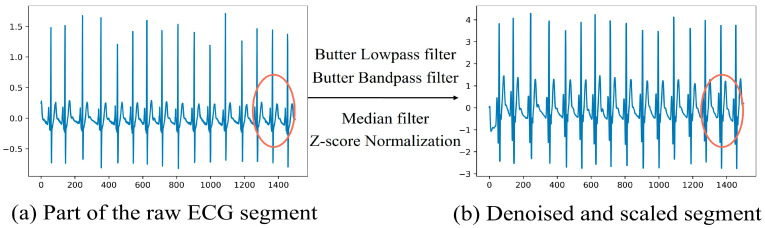
Method for the preprocessing of ECG segments. (**a**) Segment of the original ECG. (**b**) Denoised and scaled signal part.

Figure 1 illustrates the signal transformation process during preprocessing. Shown in red circle, not only does the electrocardiogram become smoother after processing, but the amplitude of the P and T waves that did not change significantly increases, making subsequent analysis easier. The samples used in this study were one-minute segments.

The samples used in this study were one-minute segments. Although the minimum duration of an OSA episode is 10 s, the probability of an OSA occurrence being split increases as the time window for segmenting the entire record is reduced. This division results in a decrease in the proportion of the sample’s effective area, thereby escalating the difficulty of OSA detection. Conversely, if the time window for sample segmentation is increased, the proportion of OSA occurrence within the effective area of the sample is similarly reduced. Consequently, this study employs a one-minute time window for data segmentation, which is strategically chosen to optimally mitigate the aforementioned issues. This time frame is considered to effectively balance the need for detailed data capture against the risk of fragmenting critical OSA episodes.

## 4. Materials and Methods

**Figure 2:**
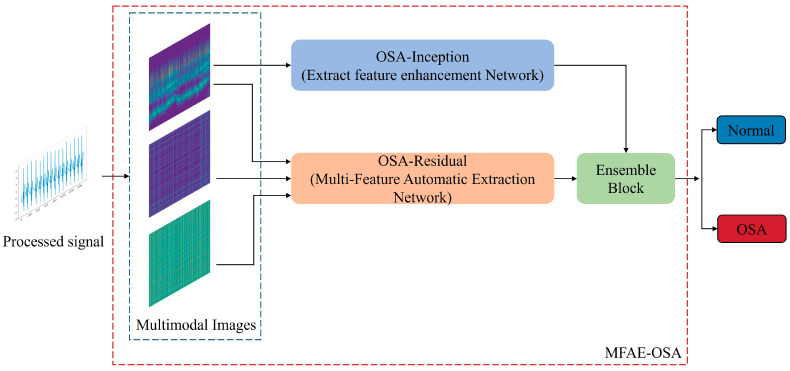
Overall structure of the proposed MFAE-OSA method. The blue dashed line represents the input multimodal images, and the red dashed line represents MFAE-OSA model.

The design of the proposed system is illustrated in Figure 2. Two hybrid datasets were created from the denoised dataset. On the preprocessed data, CWT and GAF were employed to generate the scalogram and GAF matrix image datasets, respectively. After training a composite model with a hybrid dataset, the result was obtained via a soft voting mechanism.

### 4.1. Generate Multimodal Images

Transforming electrocardiogram (ECG) signals into hybrid two-dimensional images offers considerable benefits by enriching feature diversity and capturing multidimensional data. This process allows for the visual representation of inherent temporal, frequency, and time–frequency information embedded within one-dimensional signals, thereby enhancing their feature representation. Nonetheless, this transformation introduces potential challenges. The conversion from signal to image might result in the loss of information, making the selection of appropriate conversion methodologies and parameters critical to ensure that the transformed images accurately mirror the characteristics of the original signals. Inappropriate conversion techniques may lead to the omission of essential features, underscoring the importance of choosing the correct conversion approach. Therefore, we selected the CWT and GAF as our two conversion methods.

#### 4.1.1. Scalogram Image Datasets via CWT

Time–frequency representations of signals are frequently used to interpret the information contained in physiological, linguistic, and geophysical signals. This technique is adept at discerning complex, multidimensional, and irregular signal characteristics. In contexts involving irregular data, the continuous wavelet transform (CWT) demonstrates notable proficiency [30]. Given the multifrequency nature of ECG signals, transforming them into the time–frequency domain is crucial for effective feature extraction. The CWT, widely recognized as a preeminent tool in time–frequency analysis, utilizes a spectrum of wavelet functions to dissect signals within the time–frequency domain.

CWT not only adopts, but also advances the localizing principle of the short-time Fourier transform (STFT). Unlike STFT, CWT is capable of offering high temporal resolution and low frequency resolution at higher frequencies, and conversely, high frequency resolution and low temporal resolution at lower frequencies. This is achieved through the adjustment of scale and translation parameters [31]. Consequently, scale maps generated by CWT provide a more nuanced and precise representation of signals across both low- and high-frequency domains. When applied to the irregular ECG signal, the CWT reveals a scale map with varying intensities of light and dark, thereby facilitating the clear identification of apnea signals. The CWT is expressed by Equation (2):

(2) $$C_{x}\left(s,\tau \right)=\frac{1}{\sqrt{s}}\int_{-\infty }^{\infty }x\left(t\right)\varphi *\left(\frac{t-\tau }{s}\right)dt,$$

where s represents a scale parameter, τ is a translation parameter, and φ(t) denotes the wavelet function. The scale [29] can be converted into frequency using Equation (3).

(3) $$F=\frac{F_{c}\times f_{s}}{s},$$

where $F_{c}$ represents the center frequency of φ(t), while $f_{s}$ is the sampling frequency of $x\left(t\right)$ [31].

The impact of time–frequency is dependent on the selection of the mother wavelet. We used the Morlet wavelet [32] because it closely resembles the changing trend of the ECG and is extensively applied to the analysis of ECG signals, which is defined as Equation (4):

(4) $$\varphi \left(t\right)=\frac{1}{\sqrt{b\pi }}e^{{-\left(\frac{t}{b}\right)}^{2}e^{j2\pi F_{c}t}},$$

**Figure 3:**
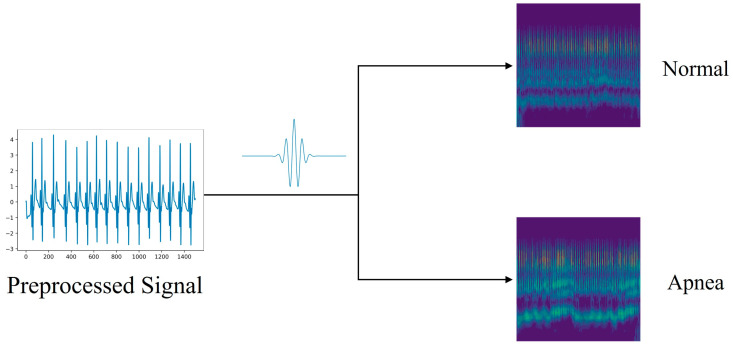
Generation process of CWT scalogram image datasets.

Parameter b is a bandwidth parameter, which is a constant in the equation. The scalogram image generation process is shown in Figure 3.

#### 4.1.2. GAF Matrix Image Datasets via GAF

Gramian angle summation field (GASF) and Gramian angle difference field (GADF) are two variants of the GAF algorithm used to encode time-series signals as images [33]. Transferring the time series to the polar coordinate space so that it has time characteristics is the central idea here.

To ascertain the scaled value $\tilde{X}$, it is necessary to ensure that the scaled signal X falls within the interval [−1, 1]. For *n* real-valued observations in a time series $x=x_{1},x_{2},\ldots ,x_{n}$, normalized from −1 to 1 as Equation (5):

(5) $$\tilde{X}=\frac{x_{i}-\max\left(x\right)+\left(x_{i}-\min\left(x\right)\right)}{\max\left(x\right)-\min\left(x\right)},$$

This method yields angular values within the [0,π] range, where the radius is equivalent to the timestamp, as described in Equation (6).

(6) $$\left\{\begin{array}{c} \phi =\arccos\left({\tilde{x}}_{i}\right),-1\leq {\tilde{x}}_{i}\leq 1,{\tilde{x}}_{i}\in \tilde{X}\\ r=\frac{t_{i}}{N},t_{i}\in N \end{array}\right.,$$

The time signature is denoted by $t_{i}$ in the above equation, and the range of the polar coordinate is standardized by a constant, N. Following the transformation into a polar coordinate system, the triangular differences between each point are employed to determine the temporal correlations over various time intervals.

The definition of the GASF and GADF is given in Equation (7).

(7) $$\left\{\begin{array}{c} GASF=\cos(\phi_{i}+\phi_{j})\\ GADF=\sin(\phi_{i}-\phi_{j}) \end{array}\right.,$$

**Figure 4:**
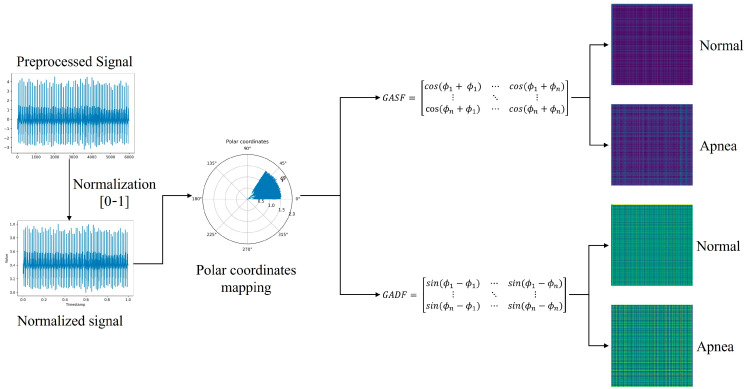
Generation process of GAF matrix image datasets.

The GAF matrix image generation process is shown in Figure 4.

Upon the creation of the image, it is essential to resize it to dimensions of (224 × 224). Figure 3 and Figure 4 display the scale maps and GAF matrix images derived from ECG segments. Figure 3 demonstrates the process of scalogram generation for the ECG, transformed utilizing the CWT technique. In contrast, Figure 4 showcases the procedure for converting ECGs into matrix images via the GAF method. As previously stated, a dataset comprising 27,282 samples for training and 6821 samples for testing was compiled.

### 4.2. Multi-Feature Automatic Extraction Network

**Figure 5:**
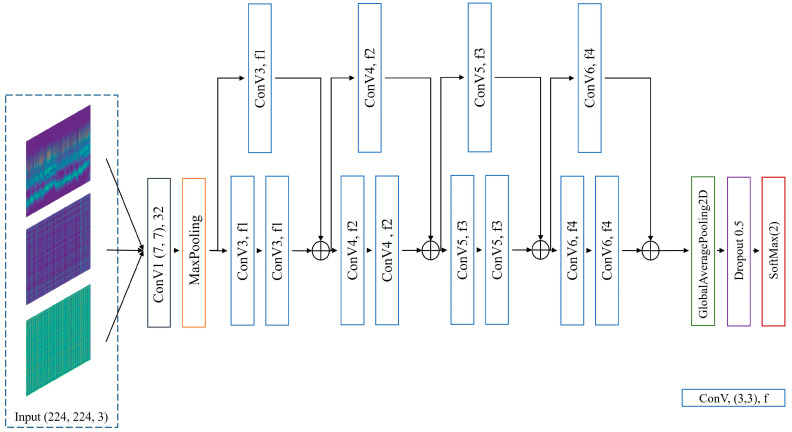
Proposed multi-feature automatic extraction network, OSA-residual model.

The ResNet [34] model, with its residual block architecture, presents numerous advantages in feature extraction. The residual learning mechanism facilitates the training of deep networks and addresses the vanishing gradient problem by allowing direct gradient propagation through skip connections. This structure enables the capture of more complex features within the data and ensures the integrity of feature information throughout the network, thereby enhancing the effectiveness of feature extraction and offering robust generalization capabilities. Consequently, when extracting features from datasets with multifaceted characteristics, our multi-feature automatic extraction network OSA-residual model is constructed based on the classical structure of residual blocks, as depicted in Figure 5.

**Figure 6:**
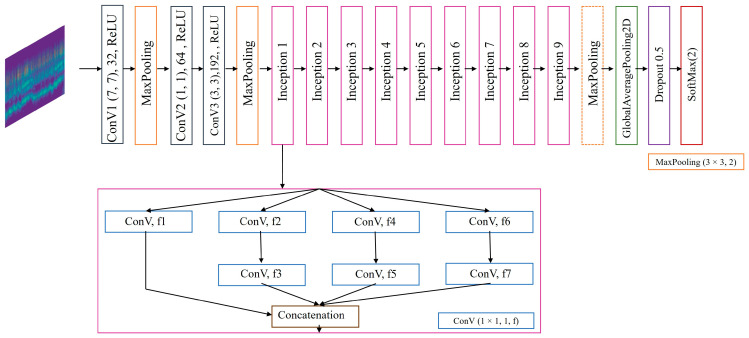
Proposed extract feature enhancement network, OSA-inception model.

The model uses an RGB three-channel image signal with a data size of 224 × 224 as the input. After 32 7 × 7 convolution filters, a ReLU activation layer, and a 3 × 3 MaxPooling, the processed signal is sent to the residual blocks, as depicted in Figure 6. The residual module is used to restore the data dimension, and a single residual module is composed of two successive convolutional layers, followed by a 1 × 1 convolutional layer used for skip connection purposes. A ReLU activation layer is added to each convolutional layer in the residual block to aggregate the feature maps from the block. Thereafter, the resultant output from each residual block is transmitted to subsequent residual blocks. There are four residual blocks with different numbers of filters in the model, and the number f of filters is 64, 128, 256, and 512 in sequence. To prevent overfitting in the model, a dropout layer with a rate of 0.5 was added. Dropout layers are employed in DNN models to reduce overfitting and decrease errors in generalization [35].

### 4.3. Extract Feature Enhancement Network

In the MFAE-OSA architecture, an extract feature enhancement network was integrated. This aims to enhance the generalization capabilities; a departure from the singular network form typically employed for feature extraction is adopted, incorporating additional network architectures. Moreover, owing to the deployment of two distinct networks for feature extraction, the resultant feature representations exhibit variance. This diversification and enhancement of features serve to enrich the overall feature landscape, contributing to a more robust and comprehensive feature-extraction process.

Should separate network enhancements be applied to each type, the overall complexity of the structure would escalate. Therefore, opting for the reinforcement of a singular type emerges as the optimal strategy. Consequently, this approach focuses exclusively on the enhancement of features derived from CWT images, which are more representative in the context of OSA diagnosis. This targeted enhancement is aimed at balancing the intricacy of the model with the efficacy of the feature-strengthening process.

The inception block [36], as a structural unit within deep learning networks, is characterized by its multiple parallel convolutional and pooling operations, enabling it to process features of varying scales concurrently. This capability allows for the capture of multi-level details in images, thereby enhancing the model’s ability to recognize features. Consequently, our extract feature enhancement network OSA-inception model was constructed based on the classical architecture of the inception block, leveraging its inherent strengths in detailed feature capture and recognition for improved model performance in OSA detection, as depicted in Figure 6.

The inception block, designed to avoid block alignment issues, confines its filter sizes to 1 × 1, 3 × 3, and 5 × 5. The 1 × 1 convolution serves a dual purpose: it reduces dimensionality and rectifies linear activation through ReLU. This structure’s modularity enables facile adjustments and expansions. OSA-Inception integrates nine inception structural blocks, encompassing convolutional, maximum pooling, dense, and supplementary layers. Sequential to the 32 convolutional filters of 7 × 7, a ReLU activation layer is employed. Figure 6 illustrates the sequential passage of the output through various inception structures.

Feature extraction from the input feature response map is accomplished in four ways: through 3 × 3 pooling and convolution kernels of three different scales: 1 × 1, 3 × 3, and 5 × 5. By amalgamating the attributes of each channel, the feature extraction process became more efficient. The specifics of the filter parameters are outlined in Table 2.

MaxPooling with dotted blocks is executed twice within the architecture, specifically between Inception 2 and Inception 3, and again between Inception 7 and Inception 8, to mitigate the risk of model overfitting. Subsequently, the output from the final residual block is directed to a 0.5 dropout layer, further safeguarding against overfitting.

### 4.4. Ensemble Block

**Figure 7:**
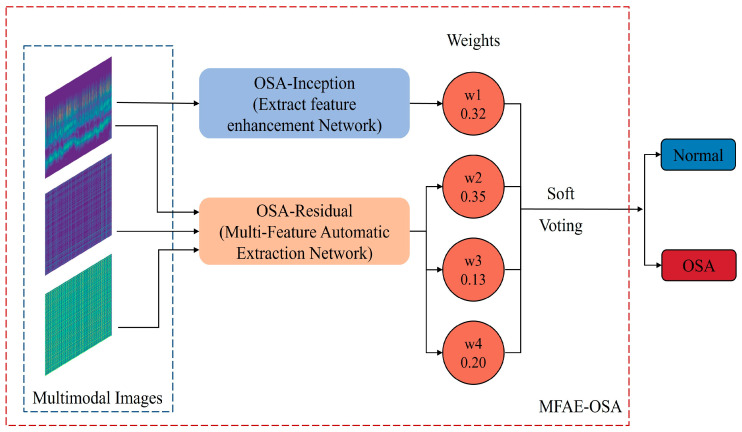
Ensemble block using soft voting structure.

Ensemble learning [37] refers to the method of integrating multiple models through a particular strategy and enhancing the precision of decision-making through group decision-making. Effective ensemble learning, in addition to requiring that the learning effect of each base learner be excellent, also requires that the difference between each base learner be as large as possible. Consequently, ensemble learning has a significant impact when combined with a model with a large variance. The performance of an ensemble learner will be superior to that of a solitary classifier. Several methods can be used to integrate learning, but this article utilizes the voting mechanism. The method of voting represents a relatively less complex approach within the realm of ensemble learning, capable of reducing the overall computational load. Hard voting, a commonly employed technique, operates on the principle of majority rule. In contrast, soft voting assigns varying weights to different models. Given the presence of four classification models in this study, the possibility of a tie scenario under hard voting is plausible. Therefore, soft voting is preferentially chosen due to its capacity to weigh the contributions of each model, thereby ensuring a more nuanced and balanced decision-making process. Equation (8) was used to determine the result, and the sum of weights for each category was computed.

(8) $$Value\left(Label=‘N’\;or\;‘A’\right)=\sum w_{i},$$

where $w_{i}$ represents the weight associated with each model’s result, as determined through empirical experimentation. Figure 7 depicts the specific weight assignment for each model.

## 5. Experiment and Result

### 5.1. Experiment Settings

Existing methods in OSA deep learning detection using single-lead ECG signals extract RR intervals and R-peak amplitudes from ECGs and use one-dimensional deep learning models for classification. In addition, the ECG time-series signal is transformed into a spectrogram and is classified using a two-dimensional deep learning model. To assess the effectiveness of the proposed approach, we selected six widely recognized models for comparative analysis: LeNet-5 [20], AlexNet [22], VGG16 [38], OSA-Residual, OSA-Inception, and LSTM [39]. The required feature method for classification with a one-dimensional deep learning model was derived from [20], with the 5 min segment replaced by a 1 min segment.

### 5.2. Performance Evaluation

The present study introduces a composite image-centric approach for the detection of OSA in one-minute segments of ECG. The effectiveness of this newly introduced method was assessed by comparing it with current techniques, using performance metrics including overall accuracy (Ac), sensitivity (Sn), specificity (Sp), and area under the curve (AUC) [40], as determined via Equations (9)–(11):

(9) $$\mathrm{Accuracy}\;=\frac{TP+TN}{TP+TN+FP+FN},$$

(10) $$\mathrm{Sensitivity}\;=\frac{TP}{TP+FN},$$

(11) $$\mathrm{Specificity}\;=\frac{TN}{TN+FP},$$

In this context, FP and TP are used to signify false positives and true positives, respectively, while FN and TN are employed to indicate false negatives and true negatives, respectively.

Additionally, given that deep learning models necessitate larger data volumes compared with other machine learning approaches, reliance on a singular dataset might prove inadequate for validation objectives. Consequently, this study employed a 10-fold cross-validation strategy [41] to affirm the reliability and robustness of the proposed model. This entailed the random partitioning of the training dataset into ten distinct subsets, with each subset serving alternately as training and validation sets.

### 5.3. Comparison of 1D CNN Model and 2D CNN Model

For comparative analysis, six standard deep learning models were utilized, with variations in the input data type. The outcomes of this comparison are presented in Table 3. Notably, due to its limited scale, the LeNet-5 model is not applicable for input data comprising CWT and GAF types in the two-dimensional CNN model; thus, it was excluded from the comparison. Additionally, the LSTM network, a typical model exemplifying temporal characteristics, was incorporated for comparison against the temporal-type data of GAF.

As depicted in Table 3, the results of the two-dimensional CNN models generally surpassed those of the one-dimensional CNN models, particularly when manually extracting features following the conversion of time series data into images. Specifically, the OSA-Inception model and OSA-Residual model demonstrated superior performance when processing a CWT time–frequency map as the input, achieving accuracies of 90.65% and 91.18%, respectively. Consequently, the OSA-Inception model was selected for feature enhancement.

Within the GAF type category, the initial three models exhibited limited effectiveness in classification. This limitation was attributed to the minimal distinction between the converted images of normal and apnea categories in the GAF series type changes, impeding these models from acquiring additional feature information through continuous learning and feature extraction. However, the OSA-Residual model network was an exception. Its unique residual structure facilitated the direct transmission of features from one layer to the subsequent layer, enabling more comprehensive feature extraction. Thus, the OSA-Residual model was capable of accurately classifying data within the GAF category. Although its efficiency was somewhat lower compared with the CWT type, it still outperformed the LSTM model, which also possessed temporal characteristics.

The two-dimensional models demonstrate a heightened capacity for extracting effective features. Moreover, the image conversion method effectively retained the feature values of the original signal to the greatest extent, minimizing errors associated with manual feature extraction. Therefore, these four models were selected for the ensemble, aligning with the study’s focus on maximizing feature extraction accuracy and efficiency.

### 5.4. Robustness Evaluation and Ablation Experiment

Given that the MFAE-OSA model encompasses multiple inputs and constitutes a composite framework, it necessitates the execution of ablation studies to substantiate its effectiveness. This involves conducting experiments on each individual module of MFAE-OSA independently, as well as incrementally adding modules for comprehensive ablation analysis. This systematic approach allows for a thorough evaluation of each component’s contribution to the overall model performance, ensuring a robust validation of the MFAE-OSA architecture.

**Figure 8:**
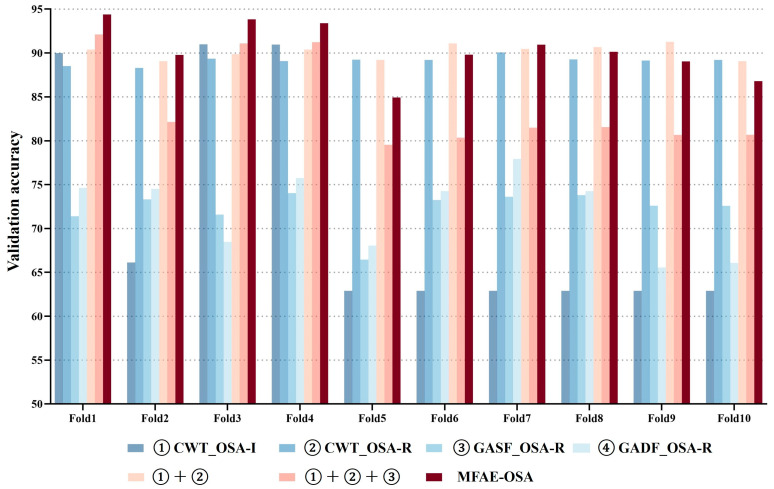
① CWT_OSA-Inception, ② CWT_OSA-Residual, ③ GASF_OSA-Residual, ④ GADF_OSA-Residual, ① + ②, and ① + ② + ③ MFAE-OSA distribution of accuracy across 10-fold cross validation. (Validation of the four ensemble models produced the best results.)

**Figure 9:**
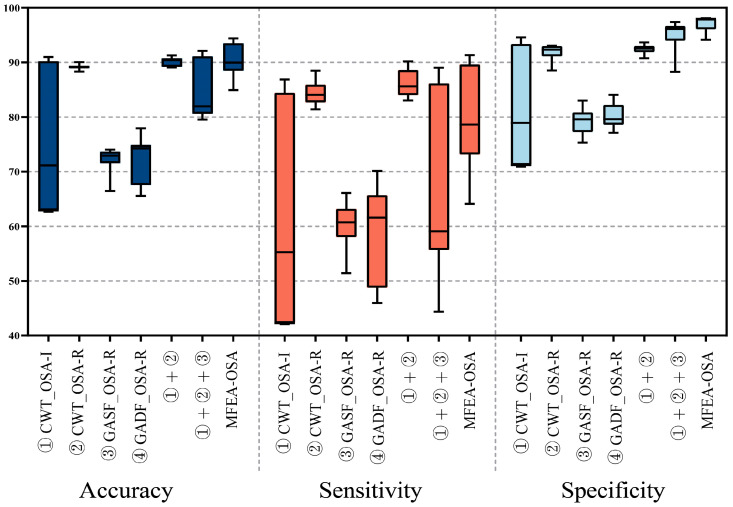
The collective results from 10-fold cross-validation for segment-based OSA detection ① CWT_OSA-Inception, ② CWT_OSA-Residual, ③ GASF_OSA-Residual, ④ GADF_OSA-Residual, ① + ②, and ① + ② + ③ MFAE-OSA.

Figure 8 illustrates the distribution of validation accuracy among the seven models, alongside the implemented 10-fold cross-validation for the proposed model. A notable divergence in the results’ variance was observed in the initial four models, aligning with the operational principles of the soft voting mechanism, which effectively leveraged large variance characteristics for optimal benefit. The comparative consistency in validation accuracy across all folds suggested the model’s robust adaptability to novel datasets. Figure 9 presents the aggregate accuracy, sensitivity, and specificity for the seven models subjected to cross-validation.

The 10-fold cross-validation demonstrated that the outcomes of the proposed model were relatively concentrated and exhibited the highest value in comparison with the other models. In the seven experimental setups, the average accuracies were recorded at 71.55%, 89.14%, 72.27%, 71.95%, 83.94%, 84.31%, and 90.31%, respectively. Correspondingly, the average sensitivities were 55.50%, 84.42%, 60.30%, 58.06%, 86.28%, 66.41%, and 79.52%, respectively.

Furthermore, the average specificities were as 78.71%, 91.92%, 79.32%, 80.14%, 94.59%, 95.18%, and 94.74%, respectively. Although the average value of the proposed model’s sensitivity was not high, it had the highest value among these performance indicators.

The efficacy of the MFAE-OSA architecture was demonstrably affirmed, with the embedded soft voting mechanism proving not only effective, but also significantly enhancing performance. This validation underscores the architectural soundness of MFAE-OSA and the strategic incorporation of the soft voting system, contributing markedly to its overall functional proficiency.

### 5.5. MFAE-OSA Model Results

In Table 4, the results of the proposed model are compared with the single best model.

**Figure 10:**
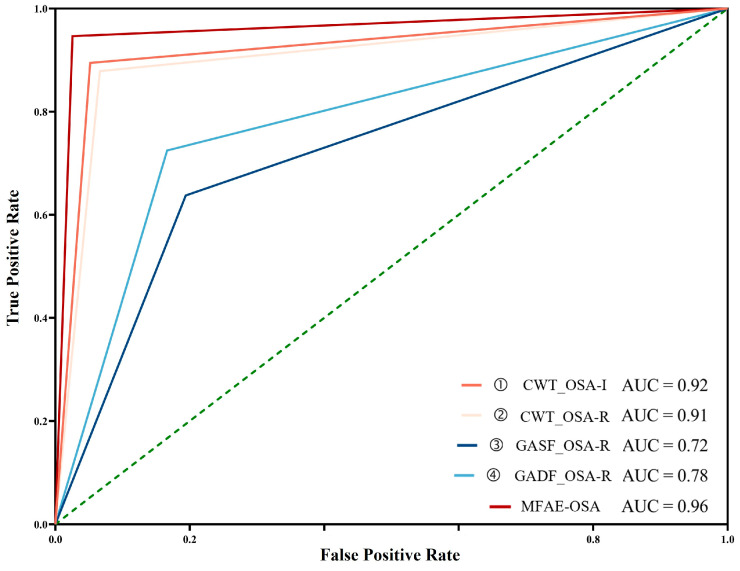
Comparison of ROC curves of ① CWT_OSA-I, ② CWT_OSA-R, ③ GASF_OSA-R, ④ GADF_OSA-R, ① + ②, ① + ② + ③, and Proposed Model in per-segment OSA detection.

The proposed model demonstrated the highest performance, achieving an accuracy of 96.37%, a sensitivity of 94.67%, and a specificity of 97.44%. Compared with other individual models, this model’s efficacy was vastly superior. Owing to its high sensitivity, the incidence of missed detection was low. In the application scenario of disease detection, it is crucial to detect the disease to the greatest extent feasible. In addition, it had a high specificity, indicating a low false-positive detection rate, which is crucial for disease detection. Sensitivity and specificity contain inherent contradictions, so they will be determined in conjunction with the ROC curve. The ROC curve is shown in Figure 10. The proposed model achieved the highest AUC score of 0.96, indicating that it had the best performance overall.

### 5.6. Comparison with Existing Methods

Given the frequent utilization of the Apnea-ECG database in apnea diagnostics, a multitude of automated obstructive sleep apnea (OSA) detection techniques are documented in the literature. In this context, the efficacy of our approach was benchmarked against other methodologies that also employ the Apnea-ECG database. Table 5 presents a comparative analysis of the per-segment OSA detection capabilities of the proposed strategy relative to various extant methods.

This demonstrates a significant performance boost using the same dataset. When using OSA-Inception and OSA-Residual to evaluate the data type with a format of CWT, our singular OSA-Inception and OSA-Residual also outperform Singh et al.’s [22] model, with accuracy increases of 6.6% and 5.11%, respectively. Wang et al.’s [20] model was 8.5% more accurate than the comparative LeNet-5 experiment in Table 3. This is because the samples used in the comparison experiment were one-minute segments, whereas Wang’s samples were five-minute segments. This demonstrates that increasing the sample size can enhance precision. Our method processes better with one-minute sample segments in disease diagnosis, as shorter sample lengths are more advantageous for real-time diagnosis. Considering that a lower false negative rate is more significant in the detection of apnea, the sensitivity of the classifier is particularly important. It was determined that our proposed model had the highest sensitivity, which was at least 2.37% higher. Moreover, our model outperformed the comparison model in terms of specificity by at least 2.64%. Although we propose that using a single GAF image type model does not work well, soft voting ensemble learning substantially improved the accuracy, sensitivity, and specificity of the final model, in addition to providing the time characteristics of the GAF image types. Compared with the original work [18], the network structure settings were modified, the input of GASF-type data was added, and the voting mechanism was modified so that the accuracy, sensitivity, and specificity increased by 5.44%, 10.99%, and 2.15% respectively. It can be seen that accuracy and sensitivity have vastly improved.

The efficacy of the MFAE-OSA algorithm presented is attributed to the synergistic integration of residual and inception architectures. The residual architecture efficiently facilitates the direct transfer of information across layers, supporting in-depth training, whereas the inception architecture adeptly captures image features at different scales, thereby augmenting the network’s expressive power and operational efficiency. The amalgamation of these frameworks results in a robust, adaptable, and efficient network configuration. This configuration enhances the capability for feature extraction, improves the flow of gradients, boosts the adaptability of the network, and maintains a balance between efficiency and performance, culminating in superior performance outcomes.

This observation indirectly corroborates the role of the diverse dataset provided in our study, which serves a complementary function in the feature extraction process. Such a dataset enhances the overall efficacy of feature extraction by integrating varied data types, thereby enriching the model’s input and potentially leading to more robust outcomes.

## 6. Discussion and Conclusions

This study aims to develop a hybrid image-based OSA detection method, integrating multimodal strategies with a composed model based on residual and inception structures. By transforming 1 min electrocardiogram segments, a hybrid image collection was generated to enhance the diversity of the dataset’s features. Our algorithm capitalizes on the diversified advantages of multi-model feature extraction to identify the incidence of OSA, outperforming existing automatic OSA detection methods in accuracy and other performance metrics. The incorporation of diverse inputs enabled the model to apprehend physiological information across multiple dimensions. HRV features delineate the cardiac response to apnea events, while temporal features indicate the chronological order of these events. This amalgamation of information provides a more enriched feature set for the identification of OSA. The features chosen are complementary, allowing the model to evaluate information from various perspectives with greater precision. Moreover, the variety in feature inputs not only bolsters the model’s capacity for generalization, but also mitigates the risk of overfitting. In essence, introducing these varied feature inputs into the model has not only elevated the precision of OSA detection.

Instead of relying on a singular feature input, this study employs a broader feature selection strategy that includes scalogram images with HRV features obtained through CWT transformation and matrix images with temporal features acquired via GAF transformation. This approach offers a wider research direction for high-precision OSA diagnosis in multi-feature input scenarios. In contrast to traditional OSA detection research, which may involve the manual extraction of fundamental features from R-wave central electrocardiogram signals and require high-level expertise, our study eschews such methods to eliminate errors from manual feature extraction and constraints due to the need for advanced skills.

In our study, we converted electrocardiogram signals into hybrid two-dimensional images, further facilitating automatic feature extraction. This provides an efficient and reliable method for OSA diagnosis. The method used for images is not limited by the duration of the signal, and the resizing feature allows for adapting the signal to a specific size. This indicates that the method exhibits high resistance to interference and transferability, even in the presence of complex signals.

Using hybrid image datasets, our model achieved 96.3% accuracy, 94.67% sensitivity, 97.44% specificity, and an AUC score of 0.96. In comparison with other studies, the proposed model had superior performance for one-minute sample classification without requiring manual feature extraction, which is dependent on the expertise and specialized knowledge of the researchers, thus helping to avert potential errors. Since our model operates on a single-lead ECG channel basis, it is applicable to wearable electronic devices or smart home medical monitoring, which is less expensive and more convenient than conventional sleep monitoring. This holds substantial importance for monitoring sleep apnea and initiating early therapeutic interventions. Furthermore, the foundation of the proposed model lies in the diagnosis of OSA through the utilization of ECG signals. As a result, ECG signals can serve the purpose of detecting other cardiovascular conditions, such as arrhythmias, conduction abnormalities, acute coronary syndromes, ventricular hypertrophy, and hypertrophy. This has several benefits for telemedicine.

Nevertheless, our methodology is subject to several limitations. Owing to the Apnea-ECG dataset’s annotation in one-minute intervals, there exists the possibility of apnea/hypopnea events spanning two such intervals, or a single interval containing multiple events. Moreover, the dataset lacks differentiation between hypopnea and apnea events in its annotations, categorizing all events under obstructive or mixed types. This limitation suggests that our proposed method might not effectively discriminate between hypopnea and apnea or detect central events. To mitigate these concerns, future research should consider employing datasets with finer temporal annotations and distinguishing among various types of respiratory pause events. Multi-modal data integration is under consideration, involving the incorporation of various data sources, such as oxygen saturation (SpO_2_), respiratory effort, and heart rate variability (HRV), along with ECG signals. Combining these data sources can provide a more holistic view of physiological changes during sleep events, enhancing the model’s capability to distinguish between hypopnea and apnea. Furthermore, the development of algorithms capable of accommodating diverse time intervals and identifying mixed-type events is crucial for enhancing generalizability. Through such enhancements, the model’s proficiency in detecting and categorizing complex respiratory events within actual clinical settings can be improved, thereby facilitating more effective diagnosis and treatment of OSA.

## Figures and Tables

**Table 1:** Parameters of single-lead ECG signals.

Parameter	Description
Signal Type	ECG signal from a single lead
Number of Signals	70
Sampling Frequency	100 Hz
Frequency Range	0.05–40 Hz
Signal Duration	420–600 min

**Table 2:** Number of filters in the 2D-inception-CNN.

Inception	$f_{1}$	$f_{2}$	$f_{3}$	$f_{4}$	$f_{5}$	$f_{6}$
Inception 1	64	96	128	16	32	32
Inception 2	128	128	256	32	64	64
Inception 3	192	96	128	16	32	32
Inception 4	160	112	224	24	46	64
Inception 5	128	128	256	24	48	64
Inception 6	112	144	288	32	64	64
Inception 7	256	160	320	32	128	128
Inception 8	256	160	320	32	128	128
Inception 9	384	192	384	48	96	64

**Table 3:** Comparison results of 1D CNN model and 2D CNN model with different input types.

Input Types	Models	Ac	Se	Sp	AUC
RR-interval R-Peaks	LeNet-5	82.43%	72.95%	88.09%	0.81
	AlexNet	85.02%	77.89%	89.28%	0.84
	VGG16	86.06%	80.19%	89.56%	0.85
	OSA-Inception	86.42%	80.95%	89.68%	0.85
	OSA-Residual	82.49%	74.12%	87.48%	0.81
	LSTM	62.70%	45.52%	71.02%	0.50
CWT	AlexNet	87.83%	83.30%	90.54%	0.87
	VGG16	90.08%	86.25%	92.37%	0.89
	OSA-Inception	90.65%	84.68%	94.21%	0.89
	OSA-Residual	91.18%	82.10%	94.55%	0.90
GASF	AlexNet	62.90%	42.28%	71.22%	0.50
	VGG16	62.90%	42.28%	71.22%	0.50
	OSA-Inception	62.90%	42.28%	71.22%	0.50
	OSA-Residual	72.23%	61.72%	78.29%	0.70
GADF	AlexNet	62.45%	45.30%	72.37%	0.50
	VGG16	62.45%	45.30%	72.37%	0.50
	OSA-Inception	62.45%	45.30%	72.37%	0.50
	OSA-Residual	75.46%	70.57%	79.68%	0.75

**Table 4:** Comparative analysis of the proposed model and the sub-model.

Input Types	Models	Ac	Se	Sp	AUC
CWT	OSA-Inception	92.80%	89.45%	94.80%	0.92
CWT	OSA-Residual	91.31%	87.87%	93.38%	0.91
GASF	OSA-Residual	74.26%	63.74%	80.59%	0.72
GADF	OSA-Residual	79.27%	72.46%	83.36%	0.78
Hybrid	MFAE-OSA	96.37%	94.67%	97.44%	0.96

**Table 5:** Comparison of the proposed approach and previous methods for segment-based OSA detection.

Reference	Method	Ac	Se	Sp
Viswabhargav et al. [17]	SVM	78.10%	78.00%	78.10%
Song et al. [6]	HMM-SVM	86.20%	82.60%	88.40%
Sharma et al. [12]	LS-SVM	83.40%	79.50%	88.40%
Kunyang et al. [18]	EDR signal NN and HMM	84.70%	88.90%	82.10%
Wang et al. [20]	LeNet-5	90.93%	83.10%	90.30%
Singh et al. [22]	CNN, Scalogram	86.20%	90.00%	83.80%
Isuru Niroshana et al. [23]	CNN, Fused images	92.40%	92.30%	92.60%
Zhou et al. [14]	Ensembled, Hybrid	90.93%	83.68%	95.29%
Proposed	OSA-Inception, CWT Scalogram	92.80%	89.45%	94.80%
	OSA-Residual, CWT Scalogram	91.31%	87.87%	93.38%
	OSA-Residual, GASF	74.26%	63.74%	80.59%
	OSA-Residual, GADF	79.27%	72.46%	83.36%
	MFAE-OSA, Hybrid	96.37%	94.67%	97.44%

## Data Availability

The PhysioNet Apnea-ECG dataset is available at http://www.physionet.org/physiobank/database/apnea-ecg/ accessed on 1 January 2024 (https://doi.org/10.13026/C23W2R).
